# Transcranial ultrasound neuromodulation of the thalamic visual pathway in a large animal model and the dose-response relationship with MR-ARFI

**DOI:** 10.1038/s41598-022-20554-4

**Published:** 2022-11-15

**Authors:** Morteza Mohammadjavadi, Ryan T. Ash, Ningrui Li, Pooja Gaur, Jan Kubanek, Yamil Saenz, Gary H. Glover, Gerald R. Popelka, Anthoney M. Norcia, Kim Butts Pauly

**Affiliations:** 1 Department of Radiology, Stanford University, Stanford, CA USA; 2 Department of Bioengineering, Stanford University, Stanford, CA USA; 3 Department of Psychology, Stanford University, Stanford, CA USA; 4 Department of Electrical Engineering, Stanford University, Stanford, CA USA; 5 Department of Biomedical Engineering, The University of Utah, Salt Lake City, Utah, USA; 6 Department of Otolaryngology, Stanford University, Stanford, CA USA; 7 Department of Psychiatry and Behavioral Sciences, Stanford University, Stanford, CA USA

**Keywords:** Visual system, Preclinical research

## Abstract

Neuromodulation of deep brain structures via transcranial ultrasound stimulation (TUS) is a promising, but still elusive approach to non-invasive treatment of brain disorders. The purpose of this study was to confirm that MR-guided TUS of the lateral geniculate nucleus (LGN) can modulate visual evoked potentials (VEPs) in the intact large animal; and to study the impact on cortical brain oscillations. The LGN on one side was identified with T2-weighted MRI in sheep (all male, n = 9). MR acoustic radiation force imaging (MR-ARFI) was used to confirm localization of the targeted area in the brain. Electroencephalographic (EEG) signals were recorded, and the visual evoked potential (VEP) peak-to-peak amplitude (N70 and P100) was calculated for each trial. Time–frequency spectral analysis was performed to elucidate the effect of TUS on cortical brain dynamics. The VEP peak-to-peak amplitude was reversibly suppressed relative to baseline during TUS. Dynamic spectral analysis demonstrated a change in cortical oscillations when TUS is paired with visual sensory input. Sonication-associated microscopic displacements, as measured by MR-ARFI, correlated with the TUS-mediated suppression of visual evoked activity. TUS non-invasively delivered to LGN can neuromodulate visual activity and oscillatory dynamics in large mammalian brains.

## Introduction

In the past decade, the biophysical and cellular mechanisms of focused ultrasound neuromodulation have become better understood^1^. Ultrasound can modulate the activity of neurons in the central nervous system by opening mechanosensitive ion channels^2–4^, which may result from changes in the fluidity of the lipid bilayer^5–8^ inducing the flux of calcium and other ions.

Transcranial ultrasound stimulation (TUS) is an extremely promising neuromodulation technology, as it can be used to noninvasively modulate neural activity in deep brain areas, which are oftentimes the most implicated in neuropsychiatric illness and inaccessible to other noninvasive neuromodulation technologies like transcranial magnetic stimulation (TMS)^9^. Although there have been several demonstrations of TUS in large animal models^10–13^, only a few have shown direct evidence of neuromodulation of deep brain areas^14,15^. Sensory systems have long been used as a test bed for studying the neuromodulatory effect of TUS. The modulation of visual evoked potentials (VEPs) by ultrasound was first shown by Fry in 1958^16^. The paper showed a transient, reversible suppression of VEPs when ultrasound stimulation was directed to the LGN of the thalamus in craniotomized cats. However, beyond the fact that the animals were craniotomized, other limitations of that study included lack of detailed information on targeting, intensity, frequency and patterning of sonication, and lack of passive or active sham stimulation conditions. More recent studies have shown ultrasound neuromodulation of visual-evoked potentials including in rodents^17^, rabbits^18^, and sheep^19^. These studies have often lacked active sham conditions limiting their generalizability, and they were targeted to sensory cortical areas which are close to the skull and therefore already accessible by TMS and do not demonstrate the true promise of TUS. No previous studies have shown VEP neuromodulation with LGN sonication in intact animals to our knowledge. We do note a recent seminal study that demonstrated robust ultrasound neuromodulation of somatosensory-evoked potentials with somatosensory thalamus sonication in pigs^20^. However, again, this was done in craniotomized animals. Overall rigorous demonstration of VEP suppression with thalamic TUS in a large animal model, with a skull highly similar to human’s, has yet to be performed.

In addition to their role in sensory evoked responses, interactions among neuronal ensembles give rise to cortical neural oscillations observable with EEG. Several mechanisms likely contribute to synchronized oscillations in the primary visual cortex in response to visual stimuli. Cortical responses in the gamma frequency range of 30–60 Hz arise from intracortical network interactions^21,22^ as well as oscillatory input from LGN of the thalamus^23^. A distinction can be made between phase-locked (PL) activity and non-phase-locked (NPL) activity^24,25^. The PL activity typically manifests as low frequency content that is consistent with the time-locked average evoked potential whereas the NPL activity contains prominent high frequency oscillations. NPL activity provides stronger evidence for the presence of synchronized or desynchronized brain cortical oscillations than PL activity^24^. To the best of our knowledge, the effect of TUS on PL and NPL oscillations has not been previously investigated.

A major barrier to translating TUS into human clinical trials is the need for in situ confirmation of targetting through the human skull which is strongly attenuating to ultrasound waves and is also highly variable across individuals^26–28^. In the case of MR-guided focused ultrasound ablation procedures, the high ultrasound intensity induces a focal and transient 15–20 °C increase in temperature that can be measured with MR-thermometry. Conversely, neuromodulation efficacy of primary sensory and motor areas can be confirmed through changes in neural activity or movements. To be able to confirm target location in areas that lack a clear sign of change and without a significant increase in temperature, other methods must be developed. Currently the most promising technique in this regard is magnetic resonance acoustic radiation force imaging (MR-ARFI), which measures the microscopic displacements generated which non-invasively measures the microscopic displacements generated by ultrasound sonication in situ, and thereby provides direct confirmation of the impact of TUS^29–31^. MR-ARFI has been demonstrated in primates^13,32,33^, but no prior study has related changes in TUS-mediated neural activity to MR-ARFI-measurements of microscopic ultrasound displacement in the same subjects.

The present work therefore sought (1) to investigate the impact of focused ultrasound targeted to the LGN through intact skull on visual-evoked activity in sheep, (2) to explore the broader effect of ultrasound directed to the LGN on cortical activity, and (3) to examine, as the first study, the relation of microscopic displacement generated by MR-ARFI (as a measure of skull attenuation), on the neuromodulation impact.

## Methods and materials

### Animal preparation and anesthesia

All procedures were approved by the Stanford Administrative Panel on Laboratory Animal Care. All experiments were performed in accordance with relevant guidelines and regulations of Stanford University. The reporting in this manuscript is in accordance with the ARRIVE guidelines^34^. Nine male sheep, 4–5 months old, 22–36 kg in body weight, were used. There were two experimental groups. An LGN TUS group consisted of 6 experiments on 5 animals; one animal underwent the experimental procedure twice with 12 days between the experiments. The sham group consisted of 5 experiments on 4 animals; one animal intentionally received zero TUS intensity, 3 animals (Active Sham) did not receive the intended TUS due to transducer dislocation caused by animal movement. One animal in Active Sham group underwent the experimental procedure twice with 3 days between the experiments, resulting in a total of 5 experiments. Active Sham Experiments in which the transducer dislocation occurred were incidentally identified at the end of the experiment by visual inspection or MRI visualization, therefore the experimenters were blind to the experimental conditions for Active Sham or TUS groups during the experiment. We do not have enough information to determine the exact location of the focal spot in the Control animals, nor do we know precisely when the transducer shift occurred during experiment. Given the physical shift of the transducer, we know that the LGN or other visual system structures most likely did not receive significant ultrasound pulses in Active Sham experiments.

All animals were anesthetized with tiletamine and zolazepam (Telazol, Lederele Parenterals, Carolina, Puerto Rico) at 4 mg/kg, intramuscularly. The anesthesia was maintained with a combination of isoflurane delivered continuously by endo-tracheal intubation and telazol delivered by intravenous infusion. Venous and arterial catheters were placed percutaneously for drug and fluid administration and blood pressure monitoring. Lactated Ringer’s solution (Abbott Laboratories, Abbott Park, IL) was administered intravenously at approximately 10 mL/kg/hr throughout anesthesia. Isoflurane and oxygen were delivered with MRI mechanical ventilation (Omni-Vent Series D, Allied Healthcare Products, St. Louis, MO) to maintain end-tidal carbon dioxide pressure between 35 and 55 mmHg. The top of the head was shaved and treated with a depilatory cream. Pulse oximetry measurements and capnography were performed continuously during anesthesia (Expression MR400, Philips Healthcare, Vantaa, Finland).

### Experiment design

**Figure 1:**
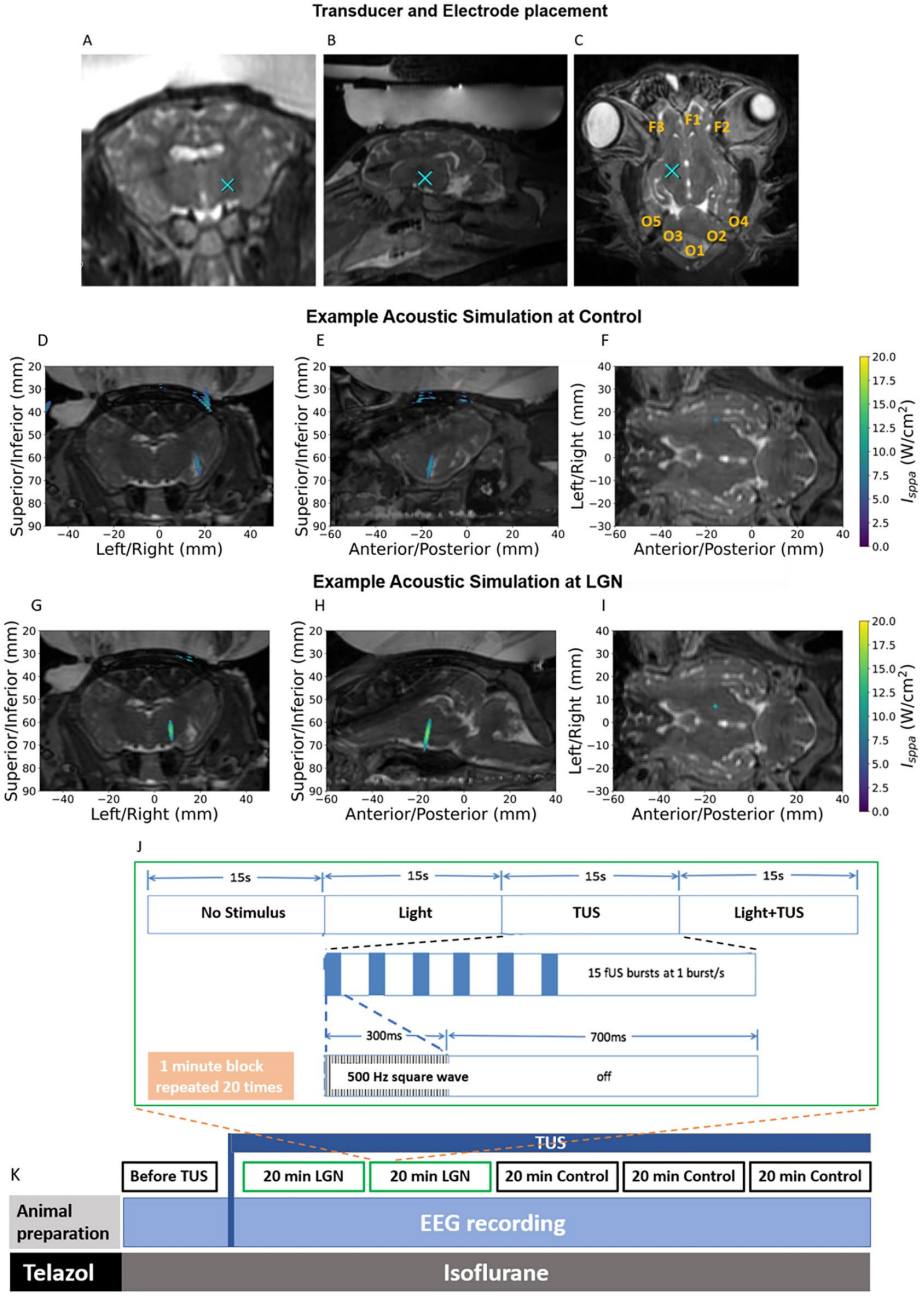
Imaging and experimental paradigm. Coronal (**A**), sagittal (**B**) and axial (**C**) MR images with TUS target location at control spot (blue X). The axial image (**C**) also shows the eight electrode locations where each electrode was extracranially placed 10 mm under the skin (orange labels). Acoustic intensities were simulated for example sonications using the hybrid angular spectrum method and overlaid on coronal (**D**), sagittal (**E**), and axial (**F**) MR images at control location, and (**G**), (**H**) and (**I**) at the LGN. In this particular example the intensity at the control location is slightly lower, however this was not always the case. Other than one experiment with substantial difference in intensity values due to a larger steering angle at control, the intensity values are comparable. Each 20-min block (**J**) was split into 20 sections, each with four 15 s conditions consisting of 1) no stimulus, 2) light stimulus only, 3) TUS-only, and 4) light stimulus-plus-TUS. For each 15-s condition the repetition frequency of the stimulus was 1 flash/s. The ultrasound stimuli were 300 ms pulse trains of square waves of 50% duty cycle, repeated every second. The visual stimuli were 20 ms white-light flashes (binocular at 1 Hz). The block diagram of the entire experiment timeline is shown (**K**). Before applying TUS, baseline VEPs were recorded for 5 min. Then the 20 min block was applied with sonication directed to either left or right LGN. This was repeated for a second 20 min block. At that point, the sonication was directed to a control non-LGN location (10 mm anterior to LGN, medial putamen and adjacent internal capsule) for three blocks. EEG was recorded continuously during each block.

Figure 1 illustrates the imaging and experimental paradigm. Coronal (Panel A), sagittal (Panel B) and axial (Panel C) MRI images show the target location at control spot (i.e., away from LGN). The axial image in (Panel C) also shows the EEG electrode locations.

Figure 1, Panels D, E and F illustrate the acoustic simulation of TUS target at the control location, and Panels G, H and I show the target at the LGN in a representative animal. The experimental stimulation paradigm with 6 blocks of data acquisition is shown in Panels J and K. The first 5-min block was a light-only stimulus condition for baseline visual activity before applying TUS. Next, there were two 20-min blocks of LGN sonication, followed by three 20-min blocks of non-LGN control spot sonication. The control site was approximately 10 mm anterior to the LGN and included a portion of the medial putamen and immediately adjacent internal capsule. Within each 20-min block there were four interleaved conditions: no stimulus (15 s), light-only (15 1 Hz stimuli), TUS-only (15 1 Hz stimuli) and light-plus-TUS (15 1 Hz stimuli). The four conditions were cycled every minute for twenty minutes. The details of the pulsing scheme are shown in Fig. 1. To establish anesthesia level in relation to VEP signal-to-noise ratio, EEG was recorded during 5-min blocks of flashing LED light only before TUS as the anesthesia lightened.

### Magnetic resonance imaging

MR image acquisitions were performed at 3 T (Signa Excite, GE Healthcare, Milwaukee, WI) using a quadrature head coil. A high resolution T2-weighted 3D FSE sequence was acquired for treatment planning with 2.5 s repetition time, 72 ms echo time, 22 cm isotropic field of view, and 256 × 192 acquisition matrix. Details of the MR imaging study are elucidated in^30^.

### VEP electrophysiology and visual stimulus presentation

Eight platinum, monopolar, 30-gauge, 10-mm long needle electrodes were implanted subdermally on the head positioned to overlie frontal (3 electrodes) and occipital (5 electrodes) cortex (Locations illustrated in Fig. 1C). The ground electrode and reference electrode were placed on the left and right ear, respectively. A 64-channel EEG amplifier system (SynAmps RT, CompumedicsNeuroScan, Australia) with 1000 Hz sampling frequency was used. The recording system was placed in a partial Faraday cage with five of the six walls covered with aluminum foil. The light stimuli were generated with white LEDs (Linrose Electronics INC, USA) embedded at the bottom of opaque paper cups placed over the closed eyes. The visual stimuli were 20 ms white-light flashes (binocular at 1 Hz).

### Transcranial ultrasound stimulation

A 1024 element, 550 kHz focused ultrasound transducer fitted with a membrane containing chilled, degassed water (ExAblate 2100, Insightec Ltd., Haifa, Israel) was affixed to the quadrature head coil, which kept it aligned in the MR space. Degassed ultrasound gel was applied to the head for acoustic coupling and the transducer placed above the head when the quadrature MR coil was connected. During the initial MRI scanning, an additional dose of xylazine (0.1–0.2 mg/kg) was administered to minimize muscle activity before EEG signal acquisition. After that, the end-tidal volume of isoflurane was kept constant (< 0.3%) during subsequent EEG data collection.

The stimulus was delivered with a fundamental frequency of 550 kHz in pulses of duration 1 ms, with a pulse repetition interval of 2 ms (pulse repetition frequency, PRF = 500 Hz), with a rectangular envelope. Each pulse train had a duration of 300 ms and a pulse train repetition interval of 1 s, with a rectangular envelope over the pulse train. Estimates of in situ ultrasound intensity were obtained retrospectively based on ex vivo fiberoptic hydrophone (Precision Acoustics, Dorset, UK) measurements of the pressure transmitted (see Supplementary Figure S1) through each skull cap. The power levels delivered during the study corresponded to in situ I_SPPA_ estimates at the target site of 19.3–63.8 W/cm^2^ (mean 46.6) and I_SPTA_ estimates of 2.8 to 9.57 W/cm^2^ (mean 6.98), corresponding to peak negative pressure ranging from 0.55 to 1.0 MPa for neuromodulation. These values were within the FDA-approved safety limit for I_SPPA_ (< 190 W/cm^2^) but not within the I_SPTA_ (< 720 mW/cm^2^) safety limit due to the high repetition rate of sonications.

#### Magnetic resonance acoustic radiation force imaging

MR-ARFI microscopic displacement was measured at a non-visual site 10–15 mm anterior to the LGN to avoid any unintended neuromodulation effects. The maximum displacement was not detectable in the region of interest of one animal, and thus was excluded from displacement measurement in Fig. 4. During MR-ARFI, focused ultrasound application spanned from the second lobe of the first bipolar through the first lobe of the second bipolar motion encoding gradient. The displacement was calculated from the complex phase difference of two acquisitions with alternating motion encoding gradient polarities using:

1 $$ D = \frac{\Delta \Phi }{{2{*}\gamma *G*\tau }} $$

where D is the average displacement (m) over the encoding period, $\Delta \Phi$ is the phase difference between two acquisitions (rad), $\gamma$ is the gyromagnetic ratio ($\frac{\gamma }{2\pi }=42.58 \text{MHz/T}$), G is the gradient magnitude ($0.04 T/m$) and τ is the duration (s) of each gradient lobe. The maximum absolute displacement in the focal spot map was found.

### Acoustic pressure and bioheat simulation

To validate targeting and estimate in situ acoustic intensities and tissue heating, transcranial acoustic propagation was simulated using the hybrid angular spectrum method^35,36^. Following treatment, skull caps were extracted from each animal, and computed tomography (CT) scans of degassed skull caps were acquired using either (1) the Artis Zeego (Siemens Healthineers, Erlangen, Germany) at 125 kVp and reconstructed with a SHARP kernel or (2) the Discovery CT750 (GE Healthcare, Waukesha, WI, USA) at 120 kVp and reconstructed with a BONEPLUS kernel. Skull caps were submerged in deionized water and degassed overnight for at least twelve hours at a vacuum pressure of 500 mmHg. After degassing, they were placed in a plastic container filled with degassed, deionized water and immediately transported from the vacuum chamber to the computed tomography scanner. The container was approximately 30 cm (width) by 60 cm (length) by 15 cm (height). The container was placed on the patient bed, and the skulls were aligned with the center of the bore. CT scans and transducer element locations were manually co-registered using T2-weighted MR images. Acoustic intensity fields were computed using a grid size of 601 by 601 by 501 at a resolution of approximately 6 points per wavelength (PPW) in the transverse directions and 18 PPW in the axial direction. Based on simulation we estimate that ultrasound intensity at the control non-LGN site was about 83% of the LGN sonication site. Tissue heating was simulated using the Pennes’ bioheat equation^37^ as implemented in the k-Wave toolbox^38^ using the acoustic and thermal properties of brain tissue specified in^39^. Bioheat simulations of LGN sonication estimated a temperature change of 1.3 ± 0.9 °C (mean + /−S.D.). Tables of simulated TUS intensities and temperature rises are provided in supplemental materials (see Supplementary Table S1 and Figure S4).

### EEG pre-processing and artifact removal

The recorded EEG signals were processed post experiment (MATLAB 2017b, Mathworks, USA). First, a band pass filter (2–50 Hz) was applied to the continuous data, then the trials from each channel were sectioned into 1 s epochs beginning 200 ms pre-stimulus and ending 800 ms post-stimulus. Automated artifact rejection was performed with EEGLAB to remove large EEG deflections^40^. Fewer than 2% of the trials were rejected after automated pre-processing. Based on visual inspection, additional epochs clearly degraded due to high noise level or a sudden change of body movement were then manually excluded. To equalize trial number across experiments, 250 trials were considered for analysis.

Independent component analysis (ICA) was then performed^40^ to remove abnormal distributions in the data associated with muscle activity. Muscle activity is noticeable as oscillations of 20–40 Hz with relatively large amplitudes^41,42^. Rejected components were identified based on the following criteria: (a) abnormal increase in frequency spectra around 20–40 Hz and higher, (b) abnormal clustered distribution on the topographic maps that resembled scalp myogenic activity, and (c) periods of abnormal power spectrum fluctuations. If the component time course showed an event related potential (ERP)-appearing signal, it was not rejected. Therefore, the ICA component removal was not automatic but a supervised process using the mentioned criteria (see Supplementary Figure S5). The main purpose of ICA analysis was to remove ultrasound artifacts from the TUS trials; therefore, ICA was not performed on Light-only conditions. Only one animal from the TUS group did not have enough EEG channels to use ICA analysis.

### Quantification of responses

#### Quantification of visual evoked potentials

For each electrode, block, and condition, the peak-to-peak amplitude (N70 & P100) was calculated from the averaged VEP. Because anesthesia increases the latency of the VEP peaks^43,44^, a time window of 40 ms was defined to identify the minimum and maximum peaks for both N70 and P100, respectively (60–100 ms time window for N70 and 90 to130 ms time window for P100). After calculating the peak-to-peak values from each electrode, the average was taken across all the electrodes. To quantify the deviation from the baseline VEP, each peak-to-peak value was normalized to the baseline condition of that animal.

### Frequency decomposition of brain oscillations

Morlet wavelet convolution^24^ was used to analyze total power, PL and NPL spectrograms of short dynamics within post-stimulus conditions. NPL power was calculated by subtracting the average evoked-related response from each trial in the time domain and then performing a time–frequency decomposition of each trial followed by averaging across spectra. Next, PL power was calculated by subtracting the NPL power from the total power. PL activity typically contains low frequency content that is consistent with the time-locked evoked potential, whereas NPL activity includes stimulus-locked oscillations such as induced beta band (13–30 Hz) or gamma band (> 30 Hz) activity. A typical example is the 40 Hz visual NPL response in human^25^.

A range of 2–55 Hz with 50 frequencies was considered for calculating the spectrum. In order to highlight temporal and frequency precision, wavelet cycles in a range of 3- to 10-cycles were used. The results were converted to decibel (dB) change relative to the −150 to 0 ms pre-stimulus baseline. Nonparametric permutation-based statistics were used to identify the significant regions of the differences between the two conditions (with no multiple comparison corrections).

## Results

### Visual evoked potentials are reversibly suppressed by sonicating thalamic LGN

**Figure 2:**
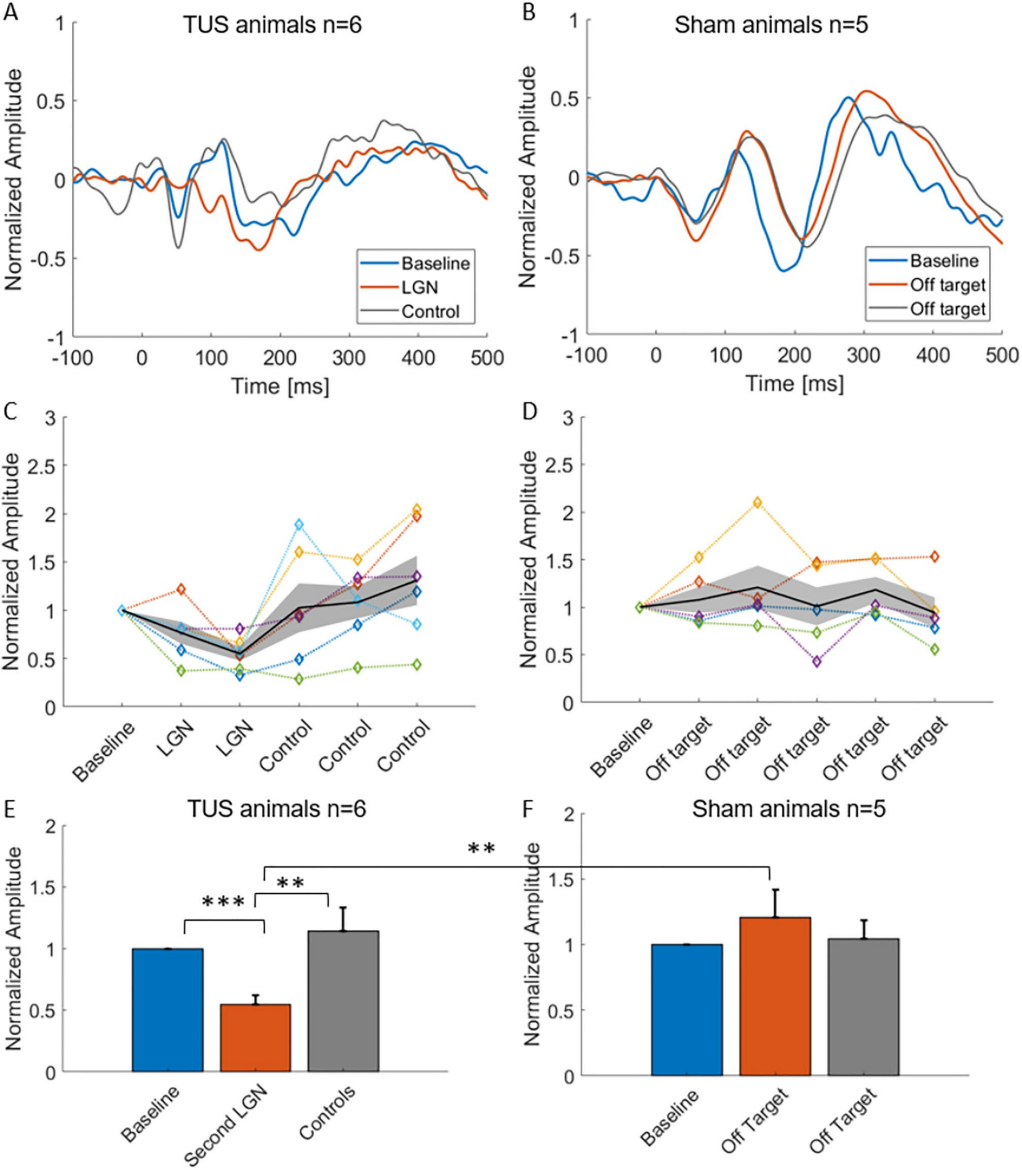
Reversible suppression of VEPs by LGN ultrasound sonication. (**A**) Grand-average VEP responses to 20-ms binocular light-flash visual stimuli before (blue line, Baseline), during (red line, LGN), and after (gray line, Control spot stimulation) sonication of the LGN. Traces were normalized to the maximal response at baseline per animal, then averaged across experiments (N = 6 experiments from 5 animals). For LGN and control VEPs, data were combined across blocks (2 for LGN and 3 for control). Only responses from the light-only condition (i.e. trials without TUS) were included in the analysis. Traces were smoothed with a 15-point (15 ms) moving averages filter for illustration purposes only. For a subset of animals, the EEG trace from -10 to + 40 ms was removed and interpolated to eliminate a light-flash-related artifact, for illustration purposes only. (**B**) Data presented as in Panel A, for the Active Sham condition (in which ultrasound sonication was determined to be off-target post-experiment, N = 4) and a no-FUS control animal (N = 1,total N = 5). (**C**) Peak-to-peak VEP amplitude measurements for individual experiments (colored lines) and Mean + /− SEM across experiments (black line), for the six experimental blocks (Baseline, two LGN sonications and three non-LGN sonications), for LGN-sonication animals. (**D**) Same as C, for Sham condition animals, across the six experimental blocks (Baseline and five off-target sonications). (**E**) Mean (+ /− SEM) VEP peak-to-peak amplitude at baseline, LGN2, and pooled control sonication blocks LGN-TUS. The mean VEP amplitude during the second LGN sonication was significantly decreased compared to Sham VEP amplitudes (**p = 0.006, ***p = 0.0007, paired one-tailed t-test, N = 6 LGN, N = 5 Sham). (**F**) Same as E, for Sham animals. All comparisons nonsignificant in F, however, there is a significance difference between center bars in (**E**) and (**F**) figures (**p = 0.008).

Figure 2 shows VEP results for the experimental conditions. Figure 2, Panel A shows mean voltage as a function of time in the LGN TUS group at baseline (blue line, prior to sonication), during LGN sonication (red line, two LGN sonication blocks combined, light-only trials), and after LGN sonication (gray line, sonication of a non-LGN location 10–15 mm anterior to the LGN including the medial putamen and adjacent internal capsule, 3 sonication blocks combined, light-only trials). The early component of the VEP, between 50 and 150 ms post-stimulus-onset, can be seen clearly in the two non-LGN sonication conditions (Baseline and Control) while the LGN sonication condition clearly suppressed the VEP. The VEP amplitude recovered after LGN sonication, during control non-LGN sonication blocks. Note that the VEPs seen here were taken from the light-only condition. This rules out the possibility that the changes in VEP were due to artifacts or non-specific auditory and somatosensory-evoked EEG responses during the application of TUS. Note that the time between experimental blocks varied from experiment to experiment, so the duration of TUS-induced VEP suppression cannot be determined with our measurements. Figure 2B shows the same data for Active Sham animals in which the sonication target was determined post-experiment to be an off-target location. In this plot there is not a clearly observable difference between sonication and baseline or post-sonication VEP amplitudes. Figure 2C shows VEP Peak-to-peak amplitude measurements in the LGN TUS group for the six experimental conditions including mean (solid black), + /−SEM (gray band) and individual data (N = 6, colored lines). Figure 2, Panel D shows the same results as in Fig. 2C but for the Active Sham group. In the LGN TUS group, sonication caused a significant decrease in response amplitude that returned to baseline on average (Fig. 2C), while Active Sham stimulation had no reliable impact on VEP amplitude (Fig. 2D). Figure 2E and F plots the mean + /− SEM VEP peak-to-peak amplitudes for the Baseline, LGN2 and Non-LGN Control blocks in the LGN TUS group (Panel E) and the Active Sham group (Panel F). The mean VEP amplitude decreased by almost 50% during LGN sonication compared to baseline and then recovered to baseline-like levels during subsequent control sonication blocks (Fig. 2E, baseline vs. LGN sonication: ***p = 0.0007; LGN sonication vs. control sonication: **p = 0.006, paired one-tailed t-test). Sham off-target sonication, in contrast, had no significant impact on VEP amplitude (Fig. 2F, all comparisons). There is a significant difference between the mean VEP amplitude during LGN sonication (Fig. 2E, center bar) compared to off-target sonication (center bar) in Fig. 2F (**p = 0.008).

**Figure 3:**
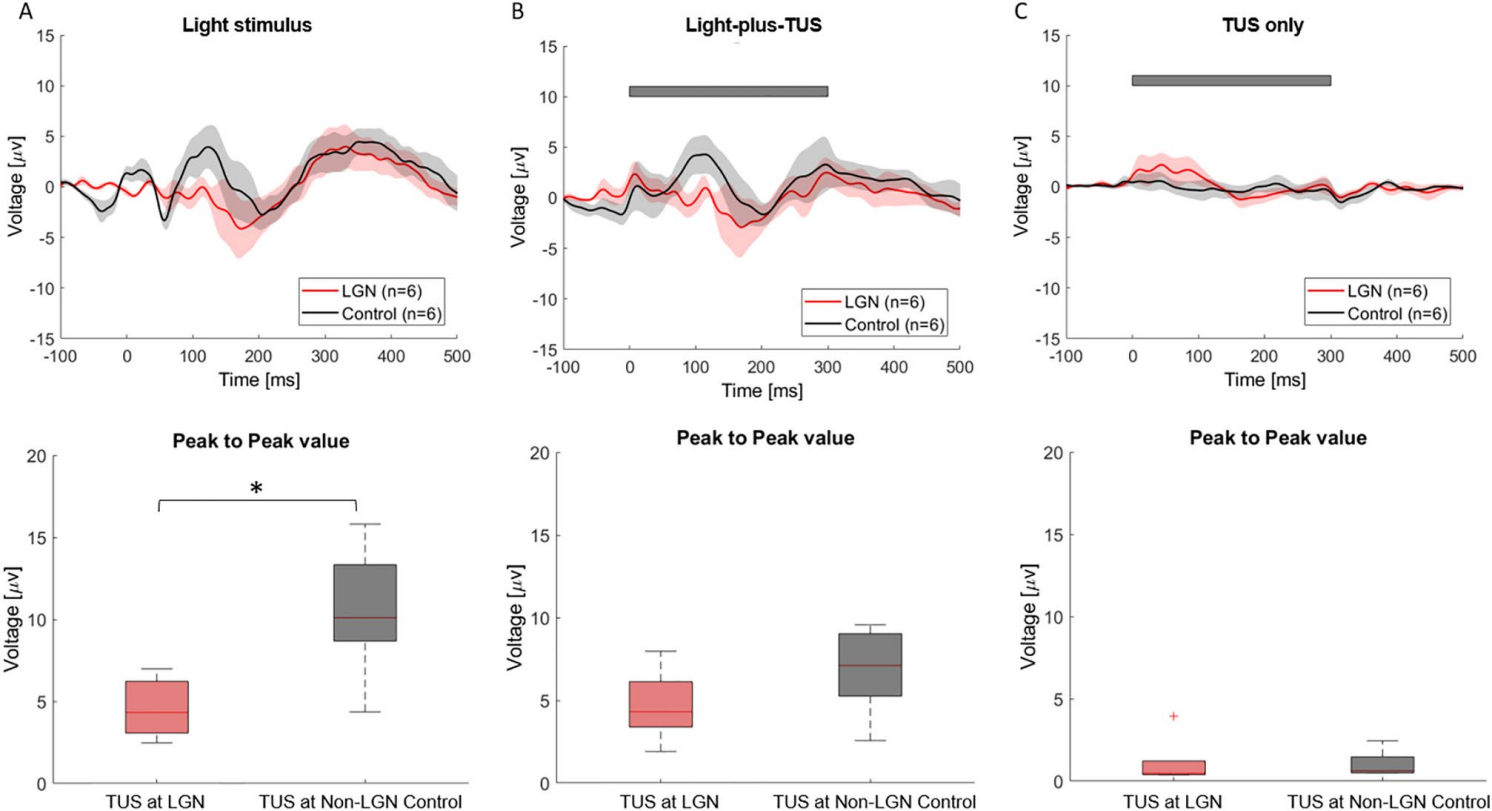
The impact of LGN TUS on VEPs outlasts the duration of sonication. Effect of LGN (red) and non-LGN control (gray, 10–15 mm anterior of LGN) sonication on VEPs for the three interleaved stimulus conditions (Light-only, Light-plus-TUS and TUS-only). (**A**) Top row: Mean + /- SEM VEP waveforms in the Light-only condition for LGN (red line, N = 6) and non-LGN control (gray line, N = 5) sonication. Responses were smoothed (15-point moving averages filter) for illustration purposes. Bottom row: Boxplots of VEP Peak-to-peak amplitude for LGN and non-LGN control sonication, light-only trials. (*p = 0.004, one-tailed unpaired t-test). Box depicts interquartile range, and dashed error bars depict full range of values. (**B**) Data presented as in A, but for the light-plus-TUS condition. Gray bar illustrates time course of TUS relative to visual stimulus onset. Difference between LGN and non-LGN: *p* = 0.07. (**C**) Data presented as in A, for the TUS-only condition (i.e., no visual stimulus). Note the minimal overall response, indicative of little-to-no nonspecific auditory or somatosensory responses to transducer vibration. The transient ~ 100 ms increase in voltage at TUS onset with LGN stimulation, is primarily due to responses in a single animal (see outlier dot in bottom row), indicative potentially of a subtle depolarizing effect of LGN-TUS in this animal, and/or an EEG artifact of TUS.

We next analyzed the immediate and lasting impact of TUS as well as the direct EEG responses to TUS without any concomitant light stimulus. Figure 3 illustrates mean VEP traces (first row) and mean response amplitudes (second row) for the Light-only (column A), Light-plus-TUS (column B) and TUS-only conditions (column C ) for the LGN (red) and non-LGN (gray) sonication conditions. These data reveal 3 essential points: (1) LGN sonication causes a VEP response suppression that is observable both during LGN sonication in light-plus-TUS trials (column B), and in light-only trials between sonication trials (column A). The impact of LGN sonication was similar in the light-only and light-plus-TUS conditions, suggesting that the major effect of sonication on VEPs is not due to an immediate impact of sonication but rather reflects a lasting suppression that outlasts the period of sonication. This is further supported by the fact that LGN sonication’s impact reaches significance in the Light-only condition (*p* = 0.004) but not the Light-plus-TUS condition (*p* = 0.07); (2) there are no observable differences in VEP waveform for the Light-only condition and Light-plus-TUS condition for the LGN or non-LGN sonication conditions, indicating that TUS by itself does not dramatically impact EEG recording quality; and (3) The EEG response in the TUS only condition is minimal. Aside from a single outlier in the LGN condition, there was no clearly observable VEP response, indicating that there is little-to-no nonspecific auditory or somatosensory-evoked responses due to application of TUS.

Given that the visual stimulus was binocular, and the LGN sonication was unilateral, visual cortical responses should decrease more ipsilateral to sonication compared to contralateral sonication. We attempted to assess the laterality of the responses by subtracting the contralateral VEPs from the ipsilateral VEPs per animal. A trend to a lateralized effect of LGN-TUS was observable. During LGN sonication, the response in the electrodes ipsilateral to stimulation were reduced by 59 ± 48% (mean + /− SD) compared to the contralateral (non-stimulated) side (*p* = 0.84, one-sample t test, n = 4), while during Control site sonication, the ipsilateral response was reduced by only 9 ± 78% compared to the non-stimulated side (*p* = 0.54). Unfortunately, due to malfunction and disconnection of a subset of electrodes in 2 experiments, only 4 animals were included in the analysis, and therefore our results were not sufficiently powered to confirm lateralization of response suppression.

### MR-ARFI displacement correlates with neurophysiological impact of TUS

**Figure 4:**
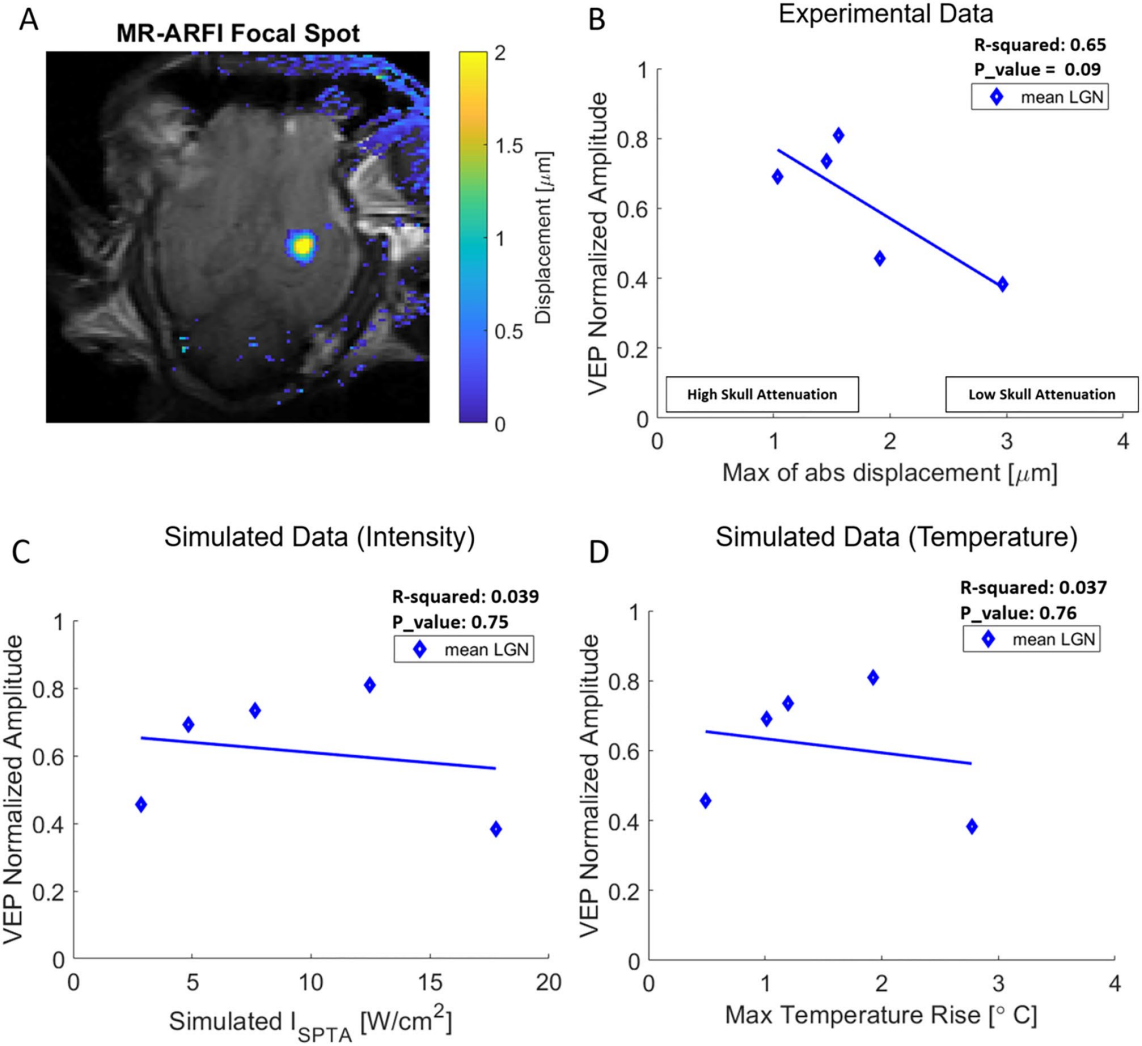
MR-ARFI displacement correlates with VEP suppression. (**A**) Representative MR-ARFI displacement at control focal spot. (**B**) Normalized peak-to-peak VEP amplitude measurements for the mean LGN (average of VEP amplitudes for LGN 1 and LGN 2) as a function of maximum MR-ARFI displacement at the focal spot. Normalized peak-to-peak VEP amplitude versus simulated intensity (**C**), and maximum temperature rise (**D**). Figures in panels C and D show no correlation between the VEP amplitude and the simulated data.

We next quantified the microscopic displacements induced by our TUS protocol. Figure 4A shows verification of the focal spot at the MR-ARFI target site in which the quantified heatmap displacement is overlayed on top of the structural MR image in a representative animal. Figure 4B plots the VEP during LGN sonication normalized to baseline per animal versus the maximum MR-ARFI displacement. Higher displacement values correlated with lower VEP peak-to-peak amplitudes (greater suppression) across subjects, indicating that MR-ARFI provides a meaningful noninvasive measure of ultrasound’s neurophysiological impact that does not depend on a significant temperature rise. The variability in microscopic displacements and therefore VEP suppression is presumably due in part to differences in skull attenuation as well as other uncontrolled experimental factors. Note that although MR-ARFI was performed at a site 10–15 mm anterior to the LGN to avoid neuromodulation that could impact later VEP measurements, the variables impacting microscopic displacement (skull attenuation, transducer coupling, etc.) are highly similar between the two sites, so measuring at the non-LGN site is a valid method to assess dose-response relationship for the LGN sonication experiments. Figure 4C and D show the VEP normalized amplitude vs the simulated I_SPTA_ and corresponding maximum temperature rise, respectively. No correlation was found between the VEP suppression and simulated intensity, nor with temperature rise at the LGN.

### Modulation of light-elicited brain oscillations by applying ultrasound to LGN

**Figure 5:**
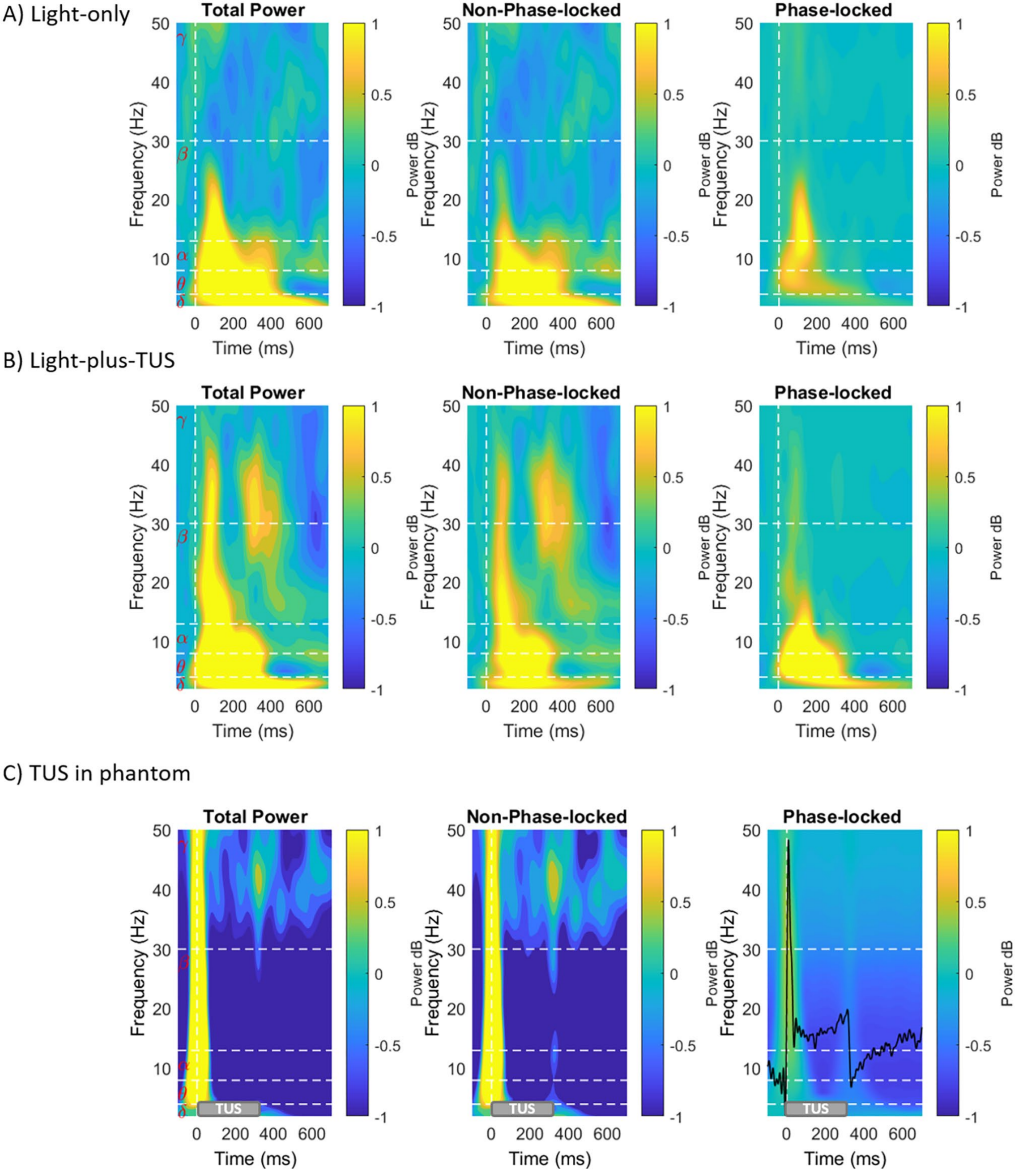
Effect of LGN TUS on Non-phase-locked and Phase-locked visual evoked cortical oscillations and EEG spectra of phantom experiments. Average (N = 5) EEG power spectra for Total Power, NPL Power, and PL Power (Columns) for two experimental conditions (Rows) (**A**) Light-only and (**B**) Light and TUS simultaneously; and (**C**) phantom experiments. Comparing Panel A with B shows the application of TUS at the LGN induces oscillations in the occipital cortex for the upper beta and gamma-band frequencies at approximately 90 and 280 ms post-stimulus. There is also a light PL activation in lower frequencies spread across theta and alpha bands. (**C**) The phantom was sonicated with 300 ms bursts at 1 Hz, while recording electrical activity (N = 5). The time domain signal plotted on top of the spectrum (black line); the gray bar shows 300 ms TUS duration. The frequency regions that differ significantly are shown in Fig. 6.

The effect of ultrasound applied to LGN on brain oscillations is shown in Fig. 5 with average spectrograms (N = 5) obtained during the light-only condition (Panel A, top row) and during the light-plus-TUS condition (Panel B, middle row). Comparing Panel A with Panel B shows that application of TUS at LGN induces NPL oscillations at upper beta and gamma-band frequencies at approximately 90 and 280 ms post-stimulus. There is also a slight PL activation in lower frequencies spread across theta and alpha bands (4–10 Hz). Increased lower band alpha power with TUS suggests that there may be less alpha desynchronization due to suppressed corticothalamic input^45^. The modulation of PL power is restricted to frequencies below 20 Hz.

Activation of the US transducer can induce an electromagnetic interference on the electrodes. The properties of the ultrasound artifact were quantified with experiments in which a rectangular block of tofu was used as a phantom with 5 EEG electrodes inserted and the ultrasound transducer placed directly on the surface. The frequency spectra of the EEG are shown in Fig. 5C with the artifact shown as a signal commensurate with the TUS signal. When the transducer was removed from the surface and positioned at increasing distances, the magnitude of the artifact decreased as the transducer distance increased. This suggests that the source of the artifact was due to electromagnetic coupling of the transducer to the electrodes.

**Figure 6:**
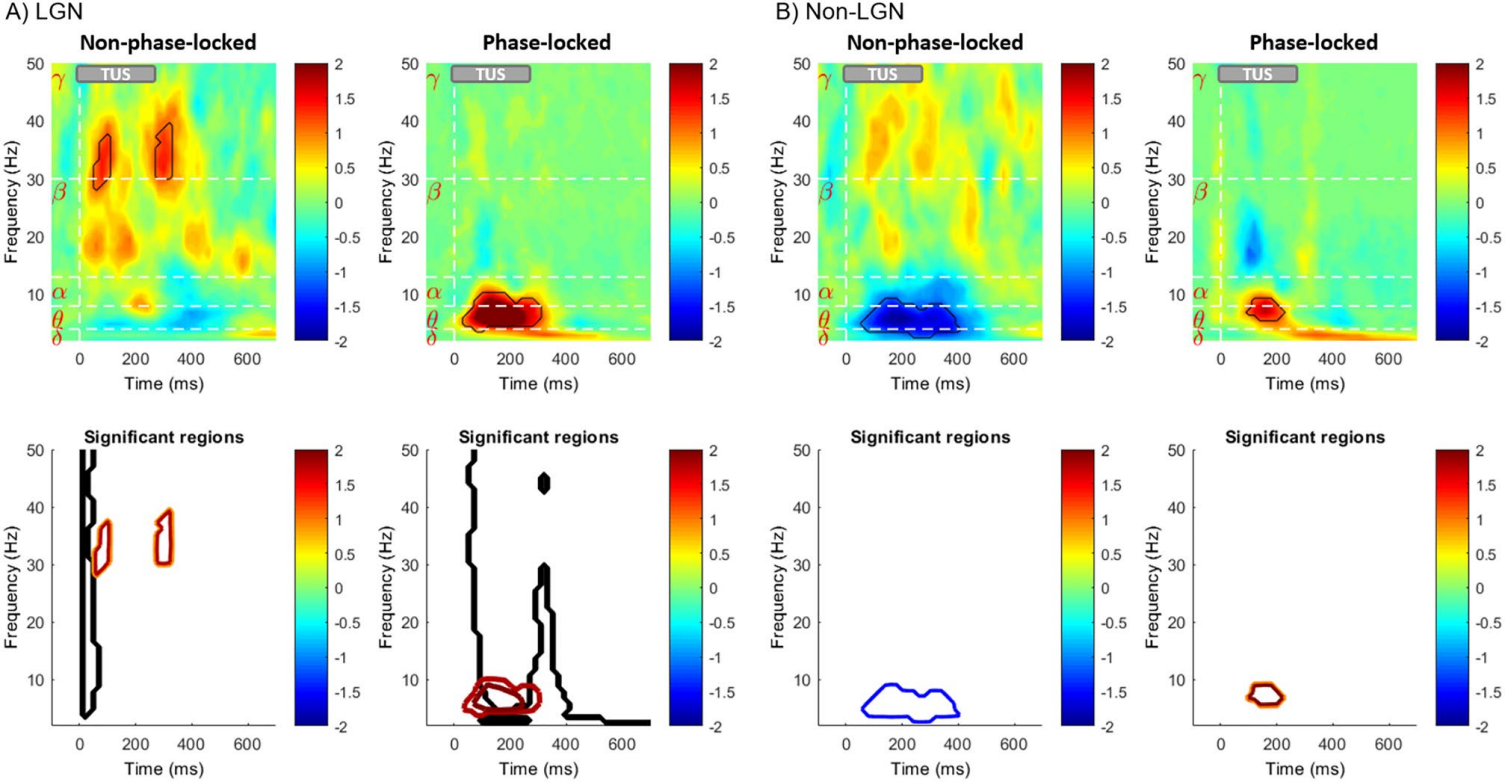
Immediate impact of LGN TUS on Non-phase-locked and Phase-locked visual evoked cortical oscillations. Differences between spectra in Fig. 5. The differences are shown as spectral difference maps (upper row) for two conditions, light only and light + TUS at LGN (Column 5A) and light only and light-plus-TUS at the non-LGN control location (Column 5B). Red coloring indicates areas where TUS increased visual-evoked power, while blue coloring indicates areas where TUS decreased visual-evoked power. Each of the two conditions contains difference maps for NPL and PL analyses. For each, the contour of significant regions based on nonparametric permutation-based statistics are shown in the bottom row of each panel (*p* < 0.05). Significant regions of spectral difference maps between tofu and light-only baseline condition are shown in black in lower row of column A. The black significant regions of Panel A are shown for only positive values.

Figure 6 illustrates differences between spectra in Fig. 5A and B. The differences are shown as spectral difference maps (upper row) for two conditions, light-only and light-plus-TUS at LGN (Column 5A) and light-only and light-plus-TUS at the non-LGN control location (Column 5B). Each of the two conditions contains difference maps for NPL and PL analyses. A nonparametric permutation-test statistic was performed (not corrected for multiple comparisons) on the difference maps between the spectrograms with differences that were statistically significant (lower row). During TUS of the LGN, significant differences in the NPL analysis were seen in the upper beta and low gamma frequency bands (30–50 Hz) at approximately 90 and 300 ms post-sonication. By contrast, in the PL analysis, significant difference was seen in the lower frequency theta and alpha bands between 90 to 300 ms. During TUS of the non-LGN control (a portion of the medial putamen and immediately adjacent internal capsule), a statistically significant reduction in the theta and alpha activity of the NPL response was observed, with no clear change in the PL response. There was no significant difference between the spectra of LGN and Non-LGN control sonication during TUS-only condition. A nonparametric statistical test was performed on the difference map between the phantom data (Fig. 5C) and the light-only baseline condition. The black contours in Fig. 6A (lower row) show the significant regions due to TUS in phantom (tofu). There is a slight overlap in low frequency (< 10 Hz) content of PL activity during the application of TUS, whereas there is no overlap in the significant regions of NPL activity, indicating that the observed effects of TUS on gamma-beta frequency powers are unlikely to be due to temporal properties of the transducer’s electromagnetic artifact.

## Discussion

We demonstrated that TUS targeted to thalamic LGN through the scalp and skull robustly and reversibly suppresses VEPs in sheep. Our VEP analysis corroborates the results of Fry et al. in which they showed a reversible reduction in the magnitude of light-elicited VEPs by over 50% with unilateral LGN sonication. The suppression was most consistent in the second LGN block suggestive of a cumulative impact of sonication, and lasted past the LGN sonication blocks in a subset of animals, indicating an enduring effect of sonication. Comparisons of EEG responses to the light-only, the light and TUS combined, and TUS-only additionally revealed that in our preparation TUS generates a minimal if any EEG response by itself, and most or all of its impact on VEP suppression is observable in interleaved light-only trials in which there is no co-occurring TUS. This rules out a number of potential TUS-related confounds, including electromagnetic TUS artifacts, auditory-evoked or somatosensory-evoked EEG responses to vibrations or sounds made by the transducer, as potential explanations for our results. We note that Lee et al.^19^ reported that sonication of primary visual cortex in sheep generates potentials at the beginning and the end of the ultrasound pulse, whereas in our study TUS of LGN did not produce a significant visual response. This suggests that cortical areas may have a more robust direct neural response to TUS than subcortical areas, although we cannot exclude the possibility that repeated LGN sonication also suppressed the response in the TUS-only condition. We note that in one animal we did see a VEP-like response in the TUS-only condition which could be similar to what Lee et al. reported although the VEP is morphologically distinct from what they reported.

Our key novel contribution is our demonstration that MR-ARFI displacement correlates with the physiological impact of ultrasound across individual animals. This argues strongly for the utility of MR-ARFI in ultrasound neuromodulation, as a way to confirm targeting in areas that lack a clear biomarker of neuromodulation (i.e. most areas outside of sensorimotor cortex) using protocols that do not cause a significant temperature rise (which is generally the case for TUS). Furthermore, we did not find any correlation between VEP normalized amplitude and simulated temperature rise.

We note that the MR-ARFI displacement map in Fig. 4a shows a larger area than the represented simulated intensity at Control in Fig. 1. The most likely reason for this disparity is that the "focal spot" on the MR-ARFI displacement map will tend to be broader than the "focal spot" on the simulated intensity field due to displacements induced by shear wave propagation away from where the acoustic radiation force is applied. Thus, the broadness of the "focal spot" on the MR-ARFI displacement map will increase with both the encoding duration and the tissue stiffness^46^. It is also possible that the simulation does not include certain factors that decrease the focality of stimulation, which the MR-ARFI does detect (such as poor coupling)–this reinforces the advantage of using MR-ARFI instead of simply using simulations. Thirdly, the relationship between ultrasound intensity and the MR-ARFI microscopic displacement may not be linearly related, which could lead to differences in the estimated sonication volume in the two modalities.

The ultrasound parameters used in this study are within the FDA-approved safety range for spatial pulse peak average (I_SPPA_) but not the spatial pulse temporal average (I_SPTA_) intensity (Supplemental materials, Table S1). It has been suggested that the FDA guidelines particularly for I_SPTA_ at frequencies less than 1 MHz are very conservative. A thorough histological examination of the brains of the sheep used in this study found no evidence of any microscopic tissue injury or damage when we sonicated for longer durations than reported in this study and over multiple days^30^, bolstering the clinical relevance of these findings.

On its face, a greater-than-50% decrease in the response to unilateral LGN sonication is larger than expected for a binocular visual stimulus, given that a single LGN should transmit at most 50% of the visual-evoked activity, and therefore a 100% inhibition should lead to an at-most 50% suppression in visual cortex. There are some speculations to explain this. First it is possible that sonication of the LGN changes the temporal dynamics of the volley to cortex, which in turn could affect the magnitude of its response. Second, other thalamic circuitry pathways particularly the reticular nucleus could be activated by TUS during the stimulation of LGN leading to more widespread inhibitory effects.

### Modulation of cortical activity through the lens of spectral analysis

In the visual system, the LGN and primary visual cortex (V1) interact in a cortico-geniculate feedback loop via the alpha-band (8–14 Hz) and through the beta-band (15–30) in geniculate-cortical feedforward processing, whereas gamma-band oscillation (> 30 Hz) is largely a property of cortex^21,23^. The origin and the functionality of these oscillations in primary visual cortex are not completely understood. To compound this further, an important body of literature exists linking the effect of volatile anesthetics to the suppression of EEG high frequency bands^47–49^. In our study, taking into account the influence of anesthesia on high frequency oscillations, we show that the upper beta-band and gamma-band activity are increased by ultrasound neuromodulation of the thalamic visual pathway. The results from simultaneous application of TUS to LGN and visual stimulation suggest that TUS of LGN amplifies visual-evoked NPL brain oscillations that occur with time and frequency characteristics analogous to the cortical oscillations elicited by visual responses^23,25^. This suggests that high frequency NPL cortical oscillations are modulated by oscillatory input from the LGN.

Legon et al.^50^ showed a time-locked gamma activity reduction around 100 ms and 200–300 ms post-stimulus-onset when sonicating the ventro-posterior lateral (VPL) of thalamus in human. In our study, the spectral results show an increase in the gamma-band activity occurring around the same time at 100 ms and 300 ms. Although the study design, species and targeted area were different in these two studies, the similarity in time of the effect and the frequency content of the oscillations caused by TUS targeted to the adjacent thalamic nuclei of the brain is worth further investigation in the future studies.

Initially there was a concern about the confounding effect of the TUS artifact on our spectral findings. Although the ICA was efficient in eliminating the artifact, as an additional control we compared the results from a phantom experiment in tofu with the results of the sheep study. The significant regions did not overlap in NPL activity, however there might be a slight overlap in slow oscillations in the PL regions. This suggests that the effects of TUS on EEG band power are not due to the TUS artifact but rather due to changes in neural activity. We also note that our main findings on VEP suppression are not confounded by sonication artifacts, as these analyses were performed on light-only (i.e. visual stimulus only) trials within the sonication blocks, in which light-only, light-plus-TUS, and TUS only trials were interleaved.

Major limitations of the current study include (1) the timing between sonication of LGN and Non-LGN control experimental blocks was variable across animals, preventing an assessment of the time-course of suppression; (2) the visual stimulus was full-field binocular not hemi-retinal and some EEG electrodes failed in a subset of animals, preventing a well-powered assessment of the laterality of the sonication’s effect; (3) the binocular light flash stimulus did not generate robust single-trial VEPs in all animals, so responses had to be averaged across multiple trials to see any effect, preventing an analysis of the build-up of sonication effects in initial trials; (4) the control sonication position was not well-defined and included both the medial putamen and a portion of the internal capsule, so the interesting effect of non-LGN sonication on cortical oscillations (Fig. 6) is difficult to interpret; (5) due to animal movement and transducer dislocation, there is not enough information to identify the location of TUS in the control animals.

Our results lend further support to the growing consensus that transcranial ultrasound neuromodulation will in the future enable noninvasive deep brain stimulation therapy for a range of neuropsychiatric illnesses, and provide proof-of-principle that MR-ARFI target confirmation will be a significant contribution to this effort.

## Conclusion

Unilateral TUS of LGN can reversibly suppress binocular VEPs in sheep. This suppression returns to baseline after termination of sonication. MR-ARFI-measured microscopic displacements during TUS predict TUS-mediated VEP suppression. Light-induced oscillations can be modulated by presenting a visual stimulus simultaneous with TUS to LGN.

## Supplementary Information

Supplementary Information.

## Data Availability

The datasets used and/or analyzed during the current study are available from the corresponding author on reasonable request.
