# Supplementary material for: Transcranial ultrasound neuromodulation of the thalamic visual pathway in a large animal model and the dose-response relationship with MR-ARFI
Source: Sci Rep. 2022 Nov 15;12:19588. doi: 10.1038/s41598-022-20554-4 (PMC9666449; doi:10.1038/s41598-022-20554-4)
Supplement: Supplementary file 1 — Supplementary Information. [file 41598_2022_20554_MOESM1_ESM.pdf]

	In Situ Estimates Using Scan Tank Measurements					Simulation			
	**Neuromodulation**			**MR-ARFI**		**Neuromodulation**			
**Experiment**	**PNP_NM (MPa)**	**ISPPA_NM (W/cm^2)**	**ISPTA_NM (W/cm^2)**	**PNP_ARFI (MPa)**	**ISPTA_ARFI (W/cm^2)**	**ISPPA_NM (W/cm^2)**	**ISPTA_NM (W/cm^2)**	**Max Temperature (C°)**	**ISPPA_Ctrl (W/cm^2)**
**1**	0.85	46.07	6.91	3.35	22.91	18.99	2.84	0.49	13.80
**2**	0.8	40.8	6.12	2.30	10.80	17.26	2.59	0.42	3.04
**3**	1.0	63.8	9.57	3.10	19.62	50.96	7.64	1.20	53.60
**4**	1.0	63.8	9.57	3.10	19.62	83.05	12.46	1.93	77.30
**5**	0.85	46.07	6.91	3.35	22.91	118.44	17.77	2.77	87.93
**6**	0.55	19.27	2.89	2.10	9.00	32.20	4.83	1.01	29.50

Table S1. Peak negative pressure and calculated intensities from in situ hydrophone measurements and simulation. The left part of the table contains the in situ hydrophone measurements in which the pressures values were measured perpendicular to the skull cap for each animal. The right side of the table are the simulated intensity values based on simulation of the actual setup from MR images. Differences between the hydrophone measurements and the simulations include differences in the location where the beam crosses the skull, the angle of incidence of the beam with the skull, and assumptions about the acoustic properties of the skull, especially in areas with low Hounsfield Units.

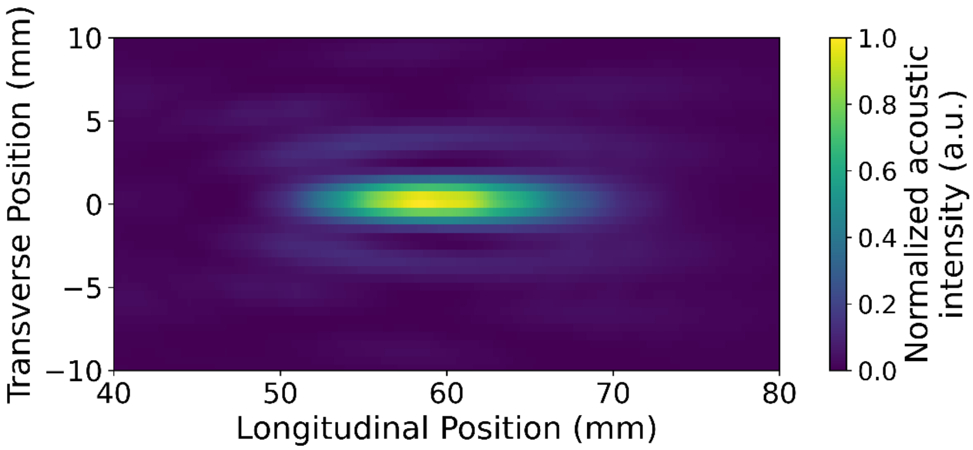

Figure S1. Hydrophone-measurement-based estimated pressure field at the focus on-axis in water. Focusing on-axis at 60 mm away from the transducer face, the full-width at half-maximum of the acoustic intensity field is approximately 3 mm in the transverse direction and 12 mm in the longitudinal direction.

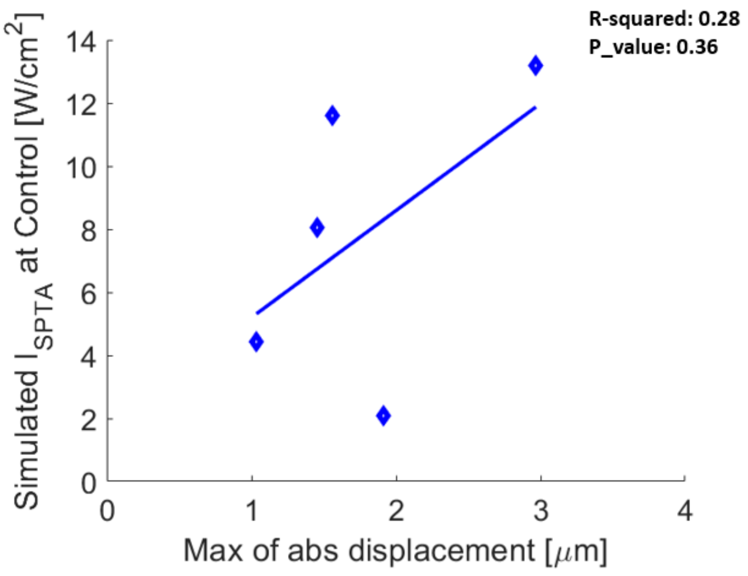

Figure S2. MR-ARFI displacement does not correlate with simulated intensity at the non-LGN control spot.

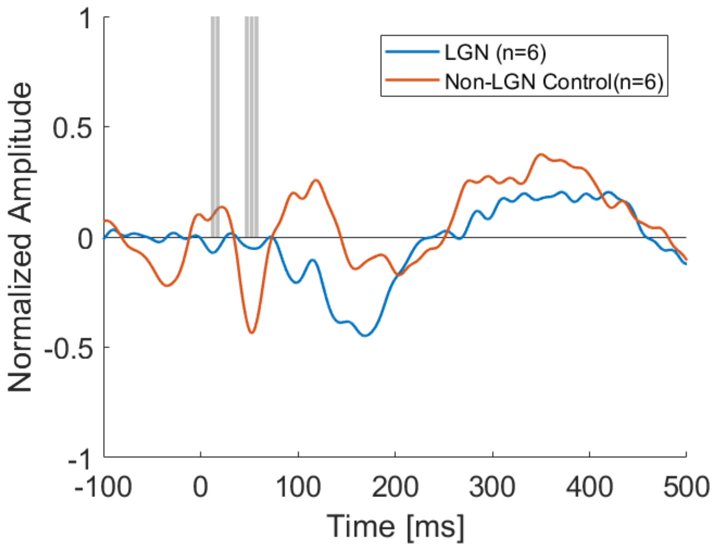

Figure S3. Statistically significant regions in the early VEP responses to light-flash visual stimuli. Shaded regions show statistically significant EEG components (5 ms bin duration, $p < 0.05$). Traces were normalized to the maximal response at baseline per animal, then averaged across experiments (N=6 experiments from 5 animals). For LGN and Non-LGN control VEPs, data were combined across blocks (2 for LGN and 3 for control).

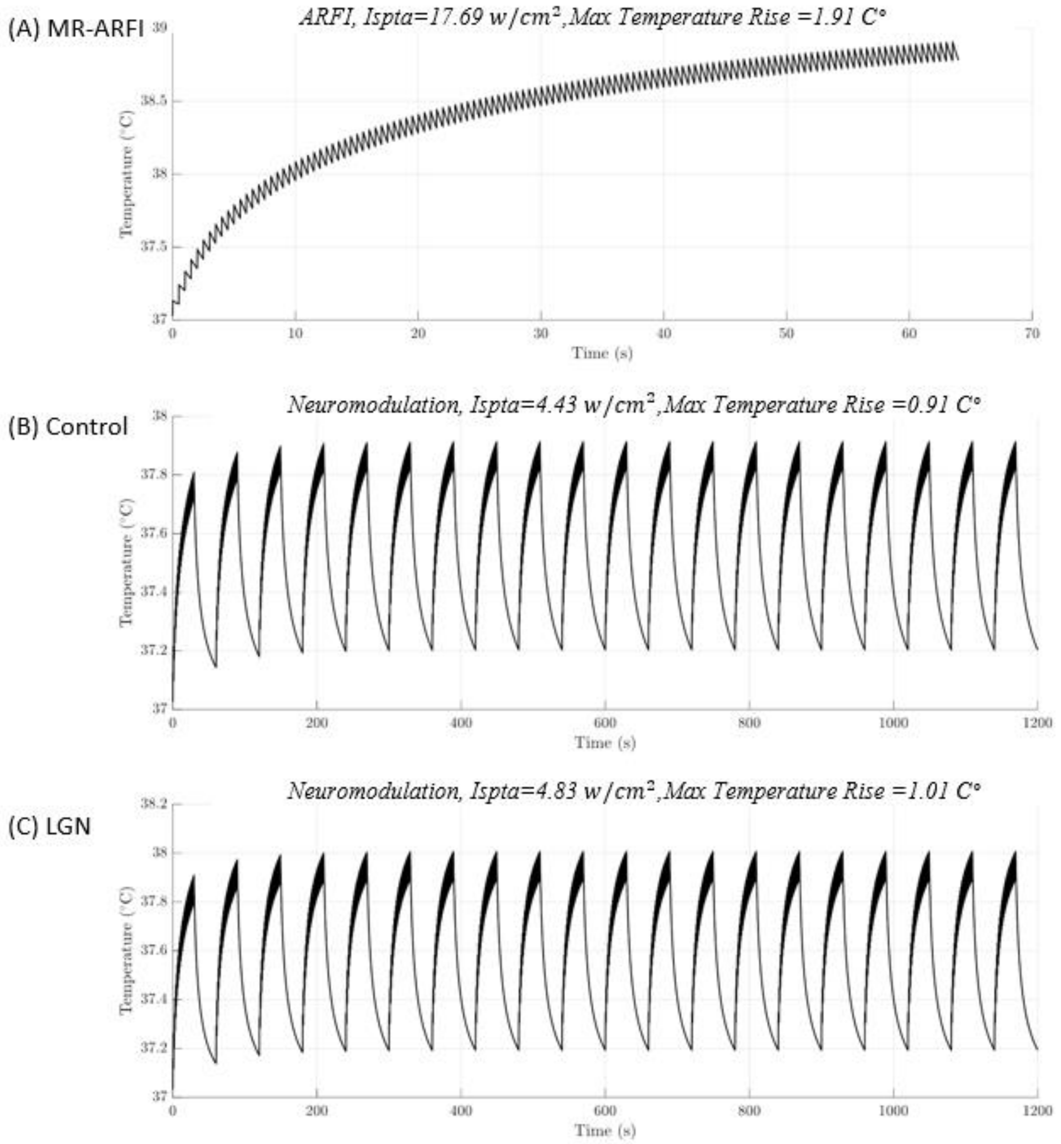

Figure S4. Representative figure of temperature rises computed from bioheat simulation. Temperature rise at MR-ARFI (**A**), Non-LGN control sonication (**B**) and LGN sonication.

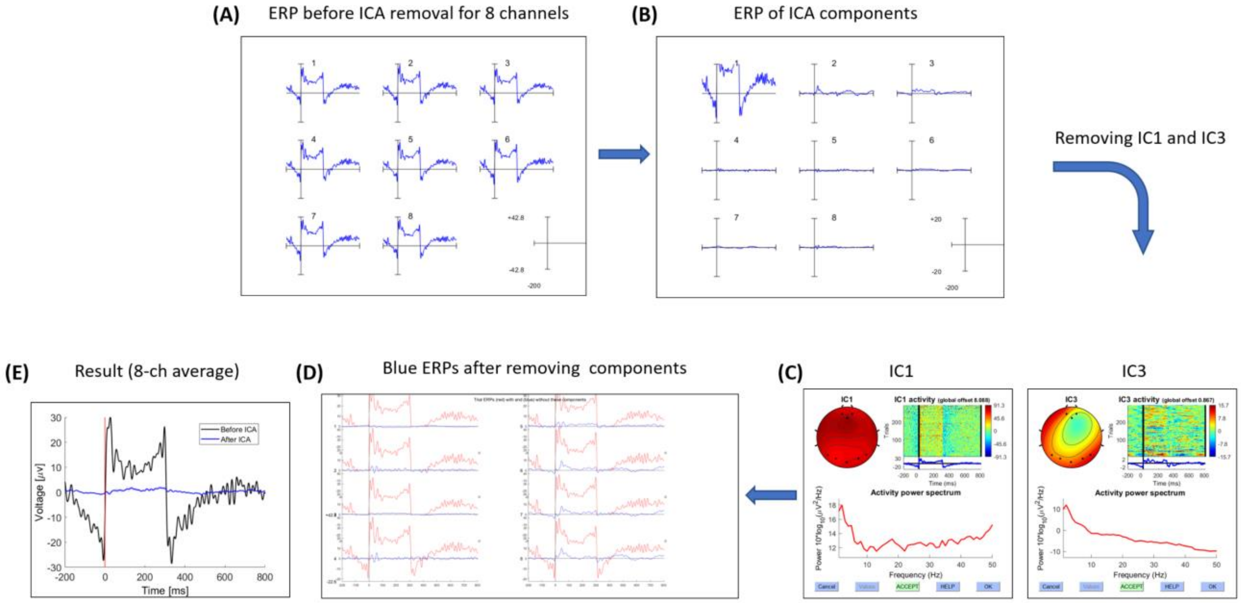

Figure S5. Processing steps for TUS artifact removal using ICA. Evoked related potentials (ERP) recorded from 8 channels in response to TUS (**A**), ERP of ICA components computed from 8 channels data using EEGlab Toolbox (**B**), determining the components that has TUS artifact-related ERP and marking them for removal, in this case components number 1 and 3 (**C**), ERP data before (red) and after (blue) removing IC1 and IC3 (**D**), this panel shows the results after taking average over 8 channels for black (Before ICA) and blue (after ICA) (**E**).
